# Supplementary material for: Systematic mapping of BCL-2 gene dependencies in cancer reveals molecular determinants of BH3 mimetic sensitivity
Source: Nat Commun. 2018 Aug 29;9:3513. doi: 10.1038/s41467-018-05815-z (PMC6115427; doi:10.1038/s41467-018-05815-z)
Supplement: Supplementary file 1 — Supplementary Information [file 41467_2018_5815_MOESM1_ESM.pdf]

Supplementary information:

Systematic mapping of BCL-2 gene dependencies in cancer reveals molecular determinants of BH3 mimetic sensitivity

Soderquist, *et. al.*

Supplementary figure 1

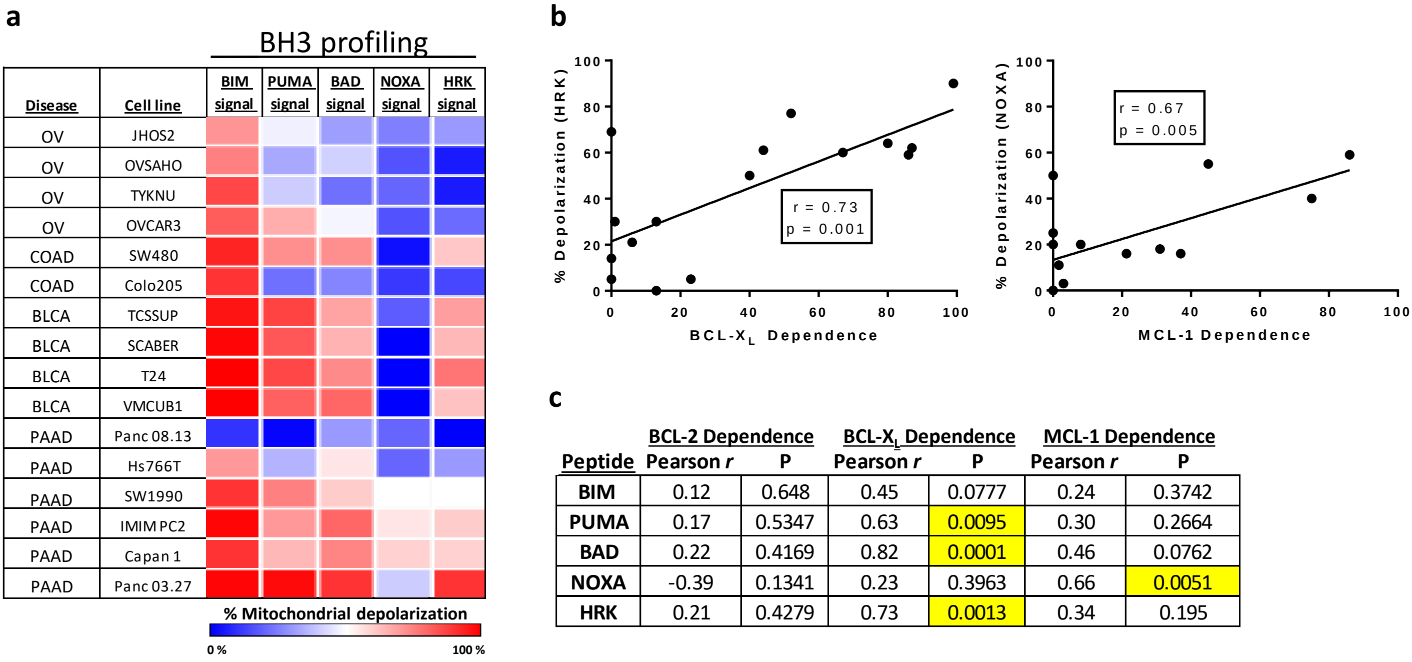

c

Peptide	BCL-2 Dependence Pearson *r*	BCL-2 Dependence P	BCL-X_L Dependence Pearson *r*	BCL-X_L Dependence P	MCL-1 Dependence Pearson *r*	MCL-1 Dependence P
**BIM**	0.12	0.648	0.45	0.0777	0.24	0.3742
**PUMA**	0.17	0.5347	0.63	0.0095	0.30	0.2664
**BAD**	0.22	0.4169	0.82	0.0001	0.46	0.0762
**NOXA**	-0.39	0.1341	0.23	0.3963	0.66	0.0051
**HRK**	0.21	0.4279	0.73	0.0013	0.34	0.195

Supplementary figure 1: BH3 profiling correlates with BH3 mimetic panel data. A, BH3 profiling was performed on selected COAD, PAAD, OV, and BLCA cell lines. BIM, PUMA, BAD, NOXA, and HRK signals were all determined using peptides from the BH3 regions of those proteins. BIM signal is a correlate for the ability of the cell line to undergo apoptosis. PUMA signal is an indicator of overall mitochondrial priming. NOXA and HRK are indicative of MCL-1 and BCL-X_L priming, respectively. BCL-2 priming can be approximated by subtracting the HRK signal (BCL-X_L priming) from the BAD signal (BCL-X_L + BCL-2 priming). **B,** Representative scatter plots are shown for select signals. **C,** Table of Pearson correlation coefficients comparing BCL-2, BCL-X_L, or MCL-1 dependence (based on percentage viability loss in cells treated with the appropriate inhibitors) to the BH3 profiling signals from the appropriate peptides. Significant correlations are highlighted in yellow.

Supplementary figure 2

a

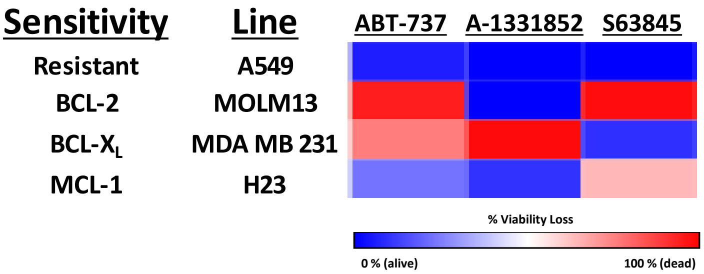

Supplemental figure 2: Additional BH3 mimetics recapitulate results from panel. A, Cell lines dependent on BCL-2 (MOLM13), BCL-X_L (MDA MB 231), MCL-1 (H23), or resistant to all tested mimetics (A549) were treated with structurally unique additional BH3 mimetics: the BCL-2/BCL-X_L dual inhibitor, ABT-737 (500 nM); the BCL-X_L inhibitor, A-1331852 (100 nM); the MCL-1 inhibitor, S63845 (100 nM). Percentage viability loss is depicted on a scale from 0% (blue) to 100% (red).

Supplementary figure 3

a

BIM induced depolarization **PUMA induced depolarization**

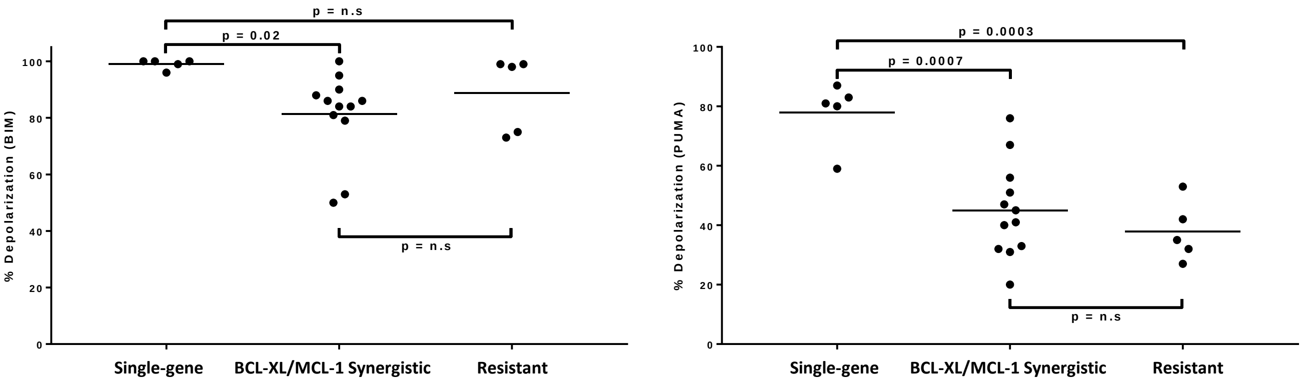

Supplemental figure 3: Comparison of BH3 profiling in cell lines based on sensitivity to BH3 mimetic panel. A, Representative cell lines that were single-gene dependent (i.e. BCL-2) (n=5), synergistically dependent on BCL-X_L + MCL-1 (n=12), or resistant to all tested combinations of mimetics (n=5) were BH3 profiled using BIM (100 µM) or PUMA (100 µM) peptides. The depolarization values for BIM or PUMA were then compared between the three groups (single-gene, synergistic, resistant) for statistically significant separation via student's t-test. The line represents the mean from each group.

Supplementary figure 4

a

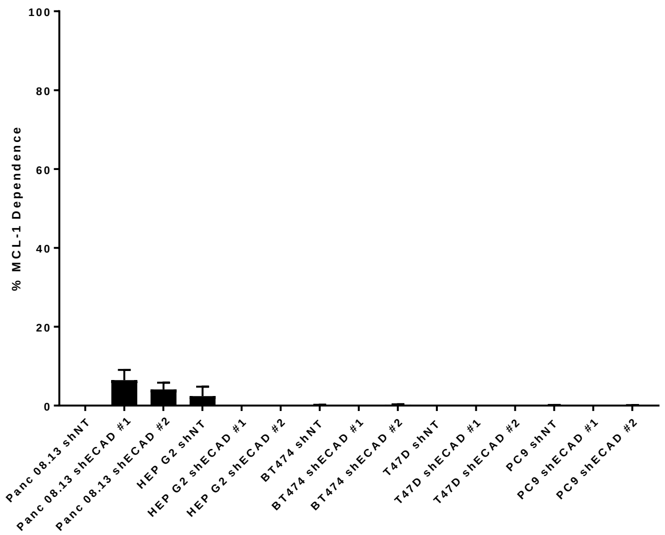

b

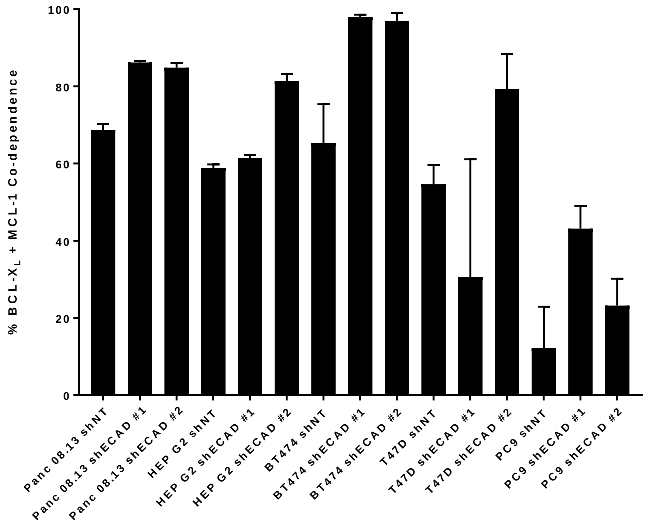

Supplemental figure 4: Driving EMT does not increase MCL-1 dependence and has only a modest impact on BCL-X_L + MCL-1 co-dependence. A, EMT was induced in selected cell lines via E-cadherin knockdown, and cells (n=3) were analyzed for MCL-1 dependence after 72 h as described in Figure 1. Data are presented as mean MCL-1 dependence +/- SEM. **B,** EMT was induced in selected cell lines via E-cadherin knockdown, and cells (n=3) were analyzed for BCL-X_L + MCL-1 dependence after 72 h as described in Figure 1. Data are presented as mean +/- SEM.

Supplementary figure 5

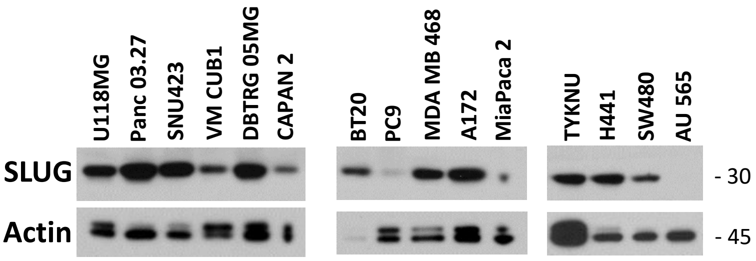

Supplementary figure 5: SLUG protein expression in select cell lines. Protein lysates were collected from 20 representative cell lines from multiple tissue types from this study. SLUG and actin were detected via western blotting for densitometry analysis. Multiple exposures of actin were used to find expression data in the linear range for densitometry.

Supplementary figure 6

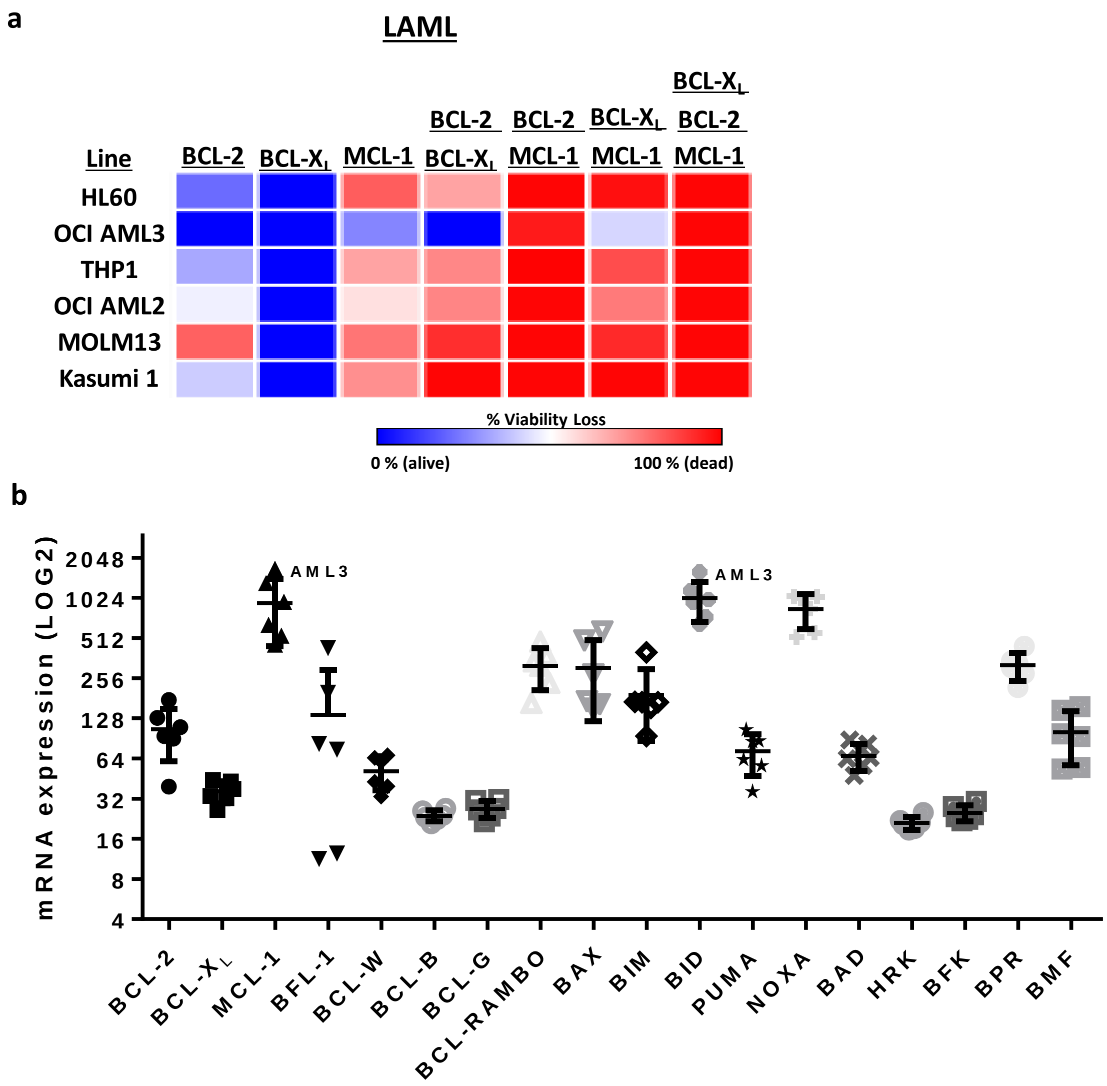

Supplementary figure 6: Overlay of BCL-2 gene expression and dependencies in LAML cell lines. A, Heatmap of BCL-2 gene dependencies in LAML. Percentage viability loss from all permutations of BCL-2, BCL-X_L and MCL-1 inhibitor combinations are depicted on a scale from 0% (blue) to 100% (red). **B,** mRNA expression data (LOG2 scores) from select BCL-2 genes from each LAML cell line. Outlier cell lines are annotated by text.

Supplementary figure 7

a

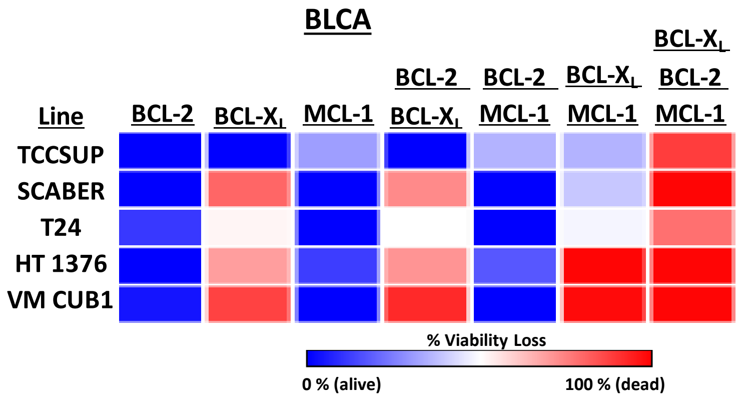

b

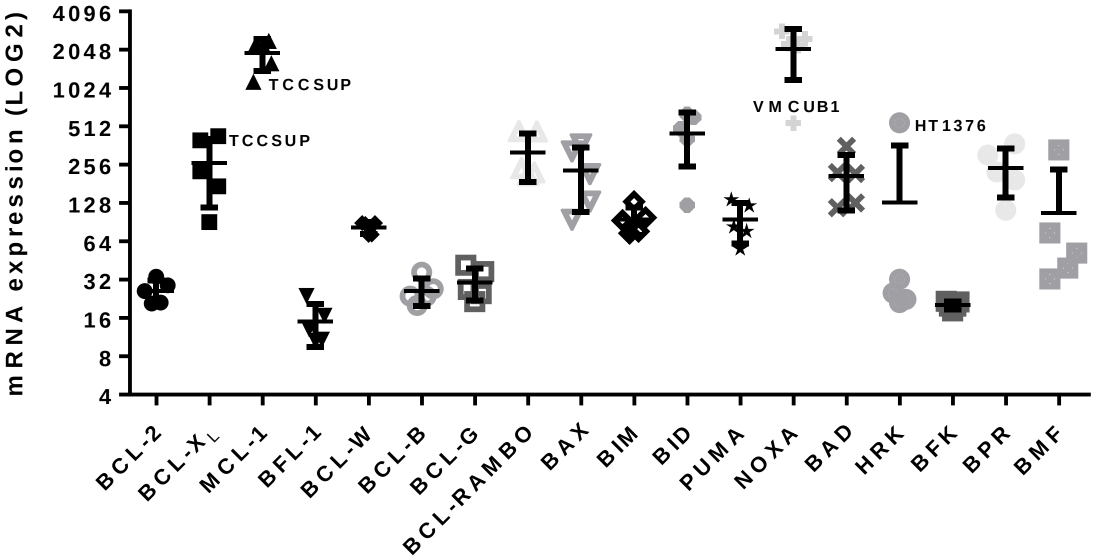

Supplementary figure 7: Overlay of BCL-2 gene expression and dependencies in BLCA cell lines. A, Heatmap of BCL-2 gene dependencies in BLCA. Percentage viability loss from all permutations of BCL-2, BCL-X_L and MCL-1 inhibitor combinations are depicted on a scale from 0% (blue) to 100% (red). **B,** mRNA expression data (LOG2 scores) from select BCL-2 genes from each BLCA cell line. Outlier cell lines are annotated by text.

Supplementary figure 8

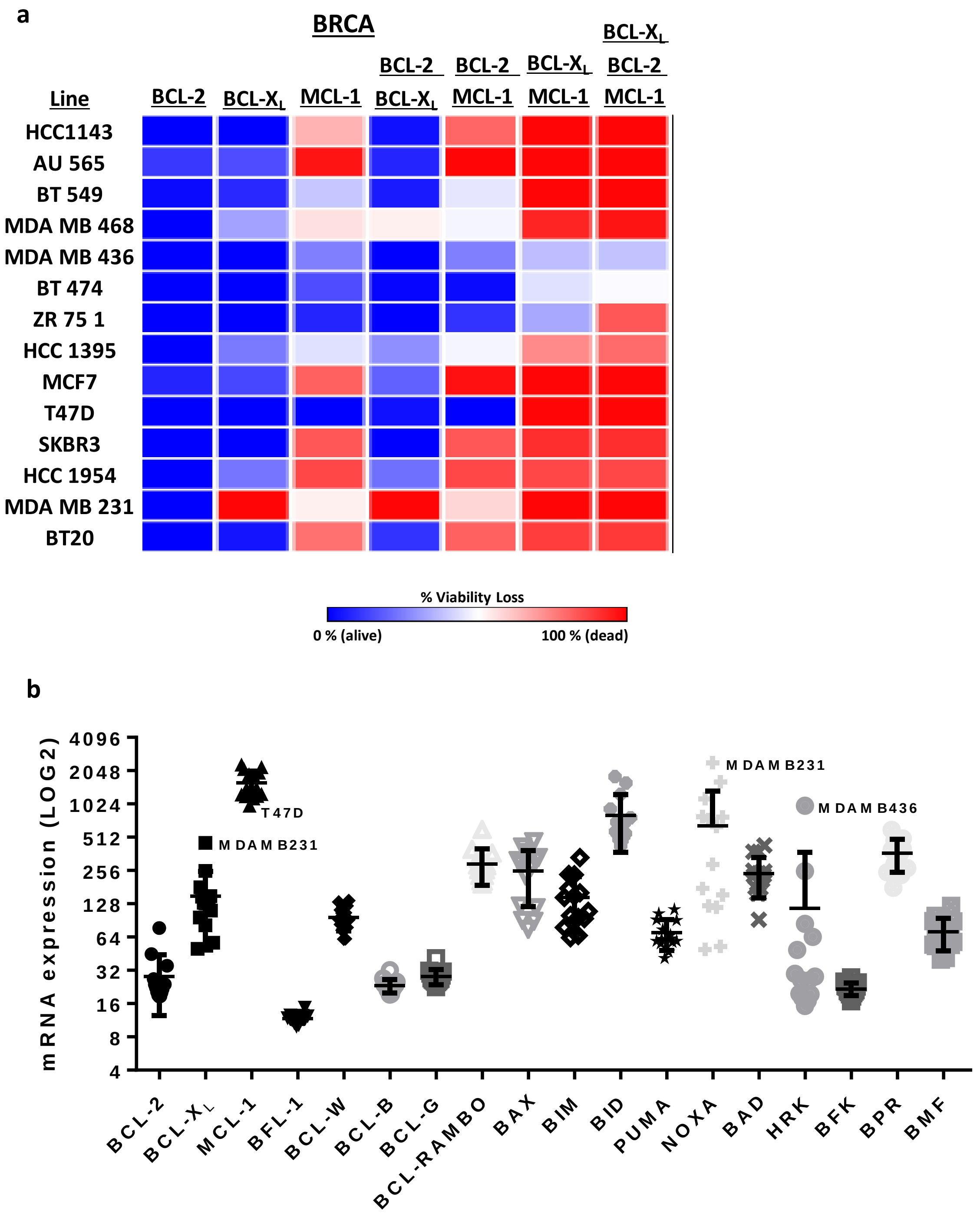

Supplementary figure 8: Overlay of BCL-2 gene expression and dependencies in BRCA cell lines. A, Heatmap of BCL-2 gene dependencies in BRCA. Percentage viability loss from all permutations of BCL-2, BCL-X_L and MCL-1 inhibitor combinations are depicted on a scale from 0% (blue) to 100% (red). **B,** mRNA expression data (LOG2 scores) from select BCL-2 genes from each BRCA cell line. Outlier cell lines are annotated by text.

Supplementary figure 9

a

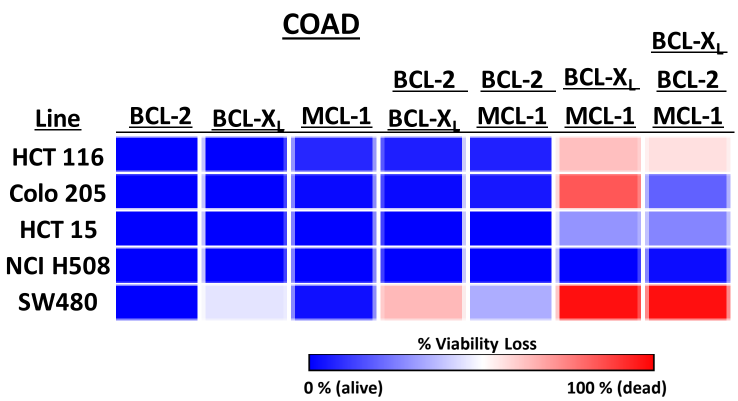

b

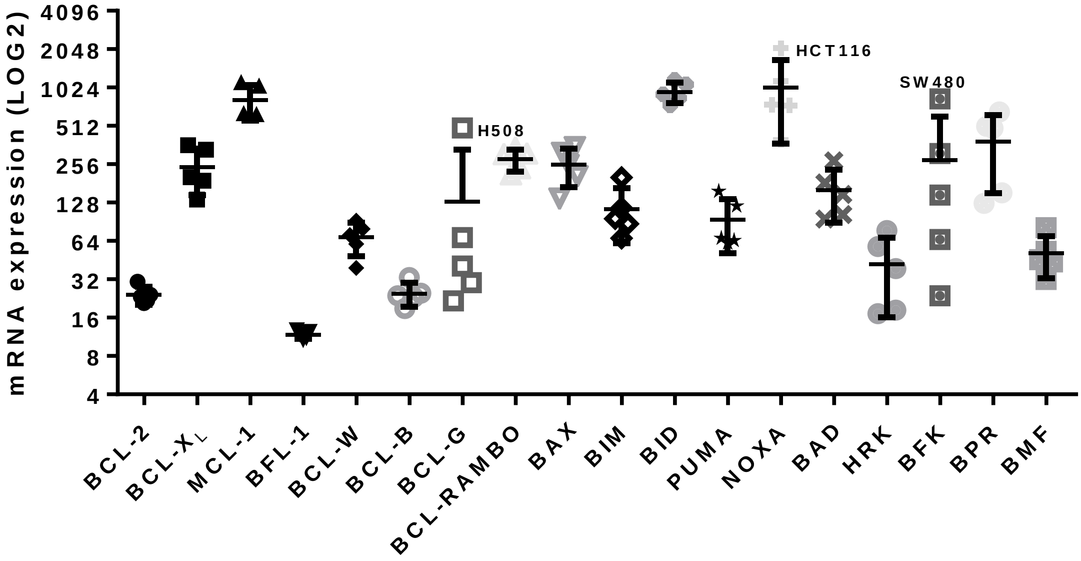

Supplementary figure 9: Overlay of BCL-2 gene expression and dependencies in COAD cell lines. A, Heatmap of BCL-2 gene dependencies in COAD. Percentage viability loss from all permutations of BCL-2, BCL-X_L and MCL-1 inhibitor combinations are depicted on a scale from 0% (blue) to 100% (red). **B,** mRNA expression data (LOG2 scores) from select BCL-2 genes from each COAD cell line. Outlier cell lines are annotated by text.

Supplementary figure 10

a

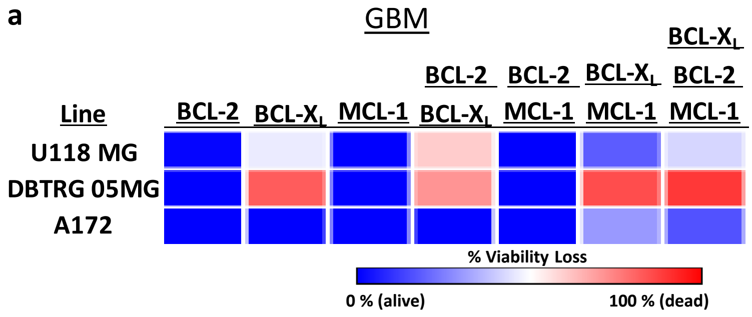

b

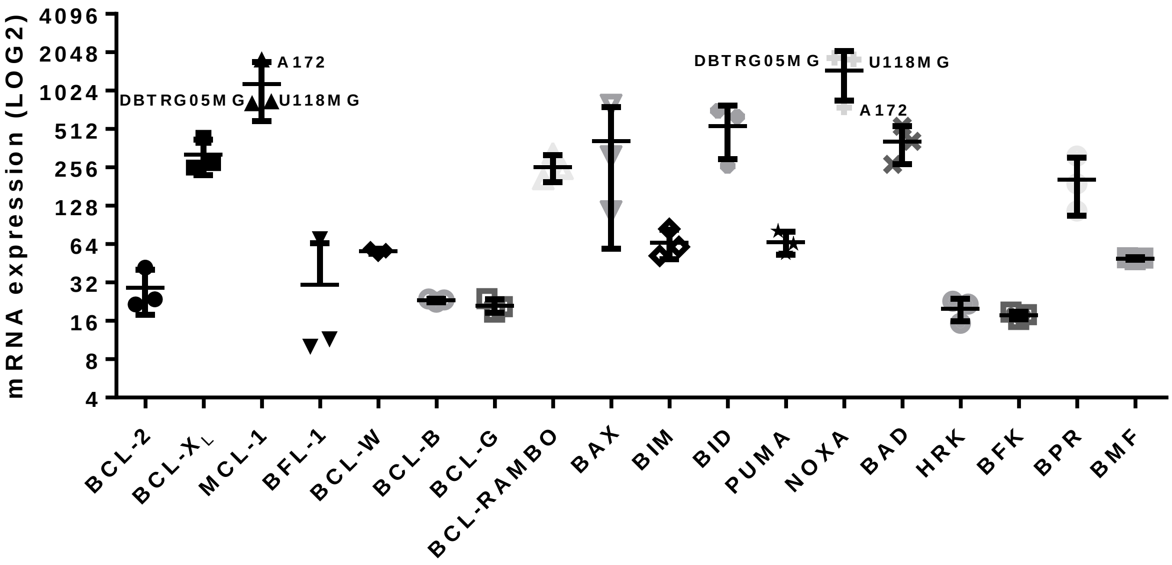

Supplementary figure 10: Overlay of BCL-2 gene expression and dependencies in GBM cell lines. A, Heatmap of BCL-2 gene dependencies in GBM. Percentage viability loss from all permutations of BCL-2, BCL-X_L and MCL-1 inhibitor combinations are depicted on a scale from 0% (blue) to 100% (red). **B,** mRNA expression data (LOG2 scores) from select BCL-2 genes from each GBM cell line. Outlier cell lines are annotated by text.

Supplementary figure 11

a

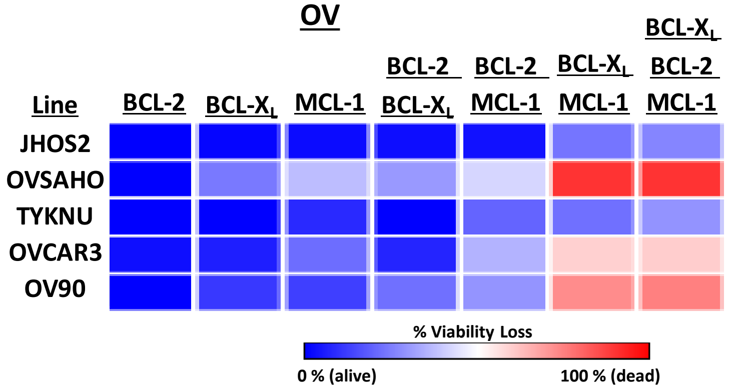

b

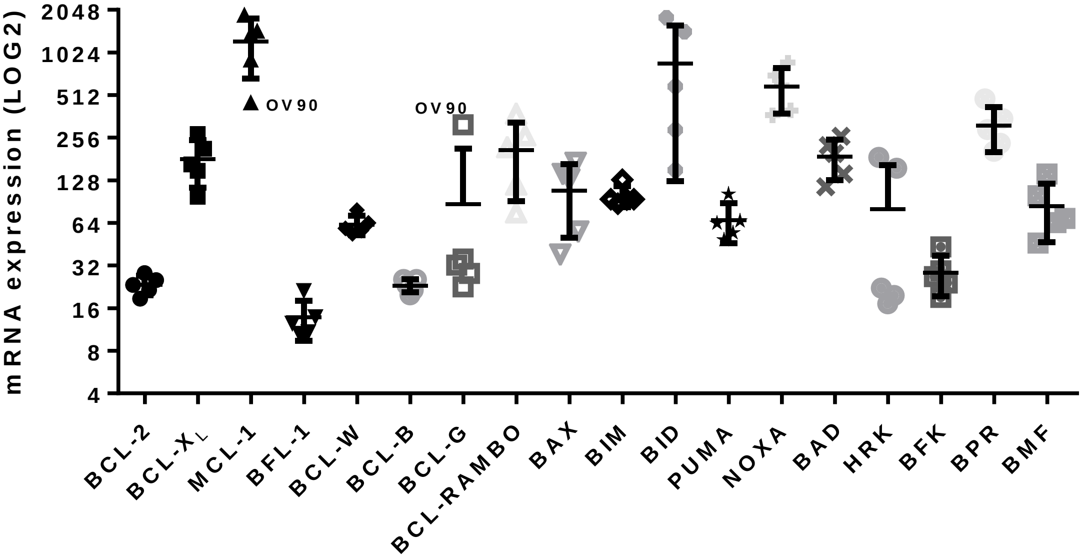

Supplementary figure 11: Overlay of BCL-2 gene expression and dependencies in OV cell lines. **A,** Heatmap of BCL-2 gene dependencies in OV. Percentage viability loss from all permutations of BCL-2, BCL-X_L and MCL-1 inhibitor combinations are depicted on a scale from 0% (blue) to 100% (red). **B,** mRNA expression data (LOG2 scores) from select BCL-2 genes from each OV cell line. Outlier cell lines are annotated by text.

Supplementary figure 12

a

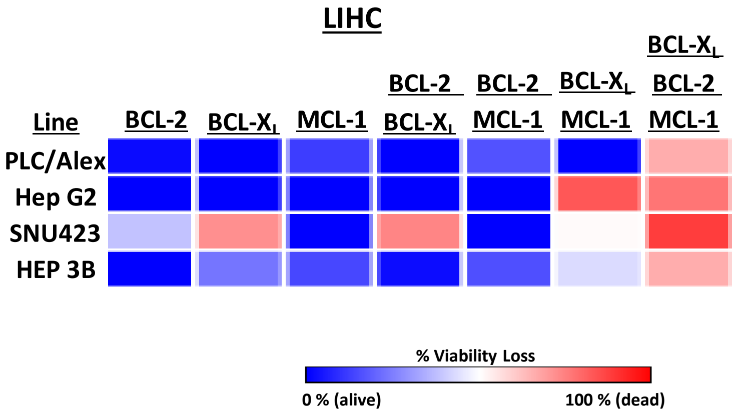

b

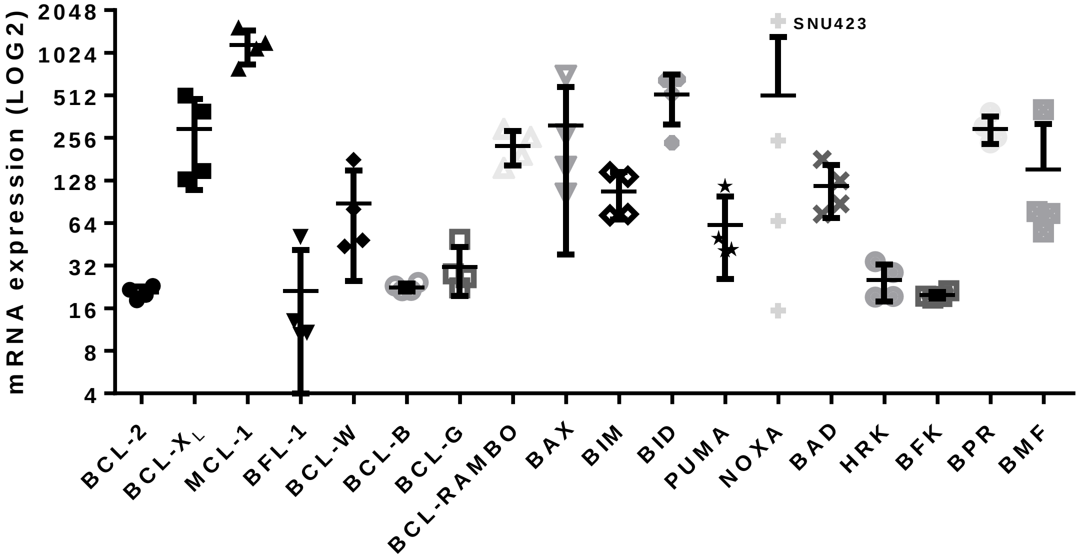

Supplementary figure 12: Overlay of BCL-2 gene expression and dependencies in LIHC cell lines. A, Heatmap of BCL-2 gene dependencies in LIHC. Percentage viability loss from all permutations of BCL-2, BCL-X_L and MCL-1 inhibitor combinations are depicted on a scale from 0% (blue) to 100% (red). **B,** mRNA expression data (LOG2 scores) from select BCL-2 genes from each LIHC cancer cell line. Outlier cell lines are annotated by text.

Supplementary figure 13

a

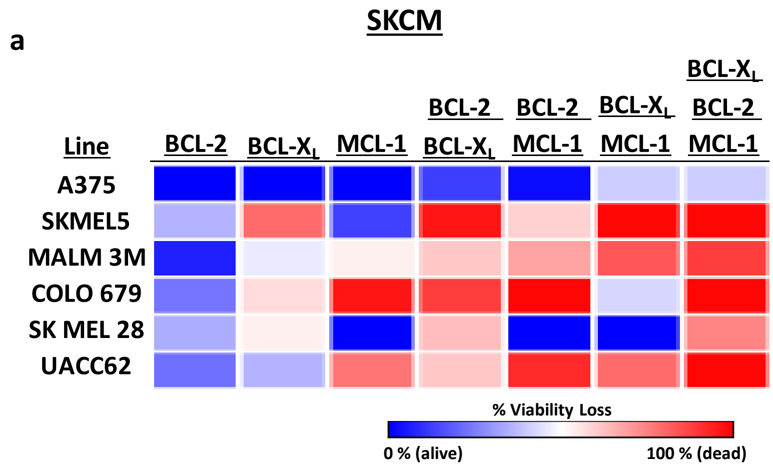

b

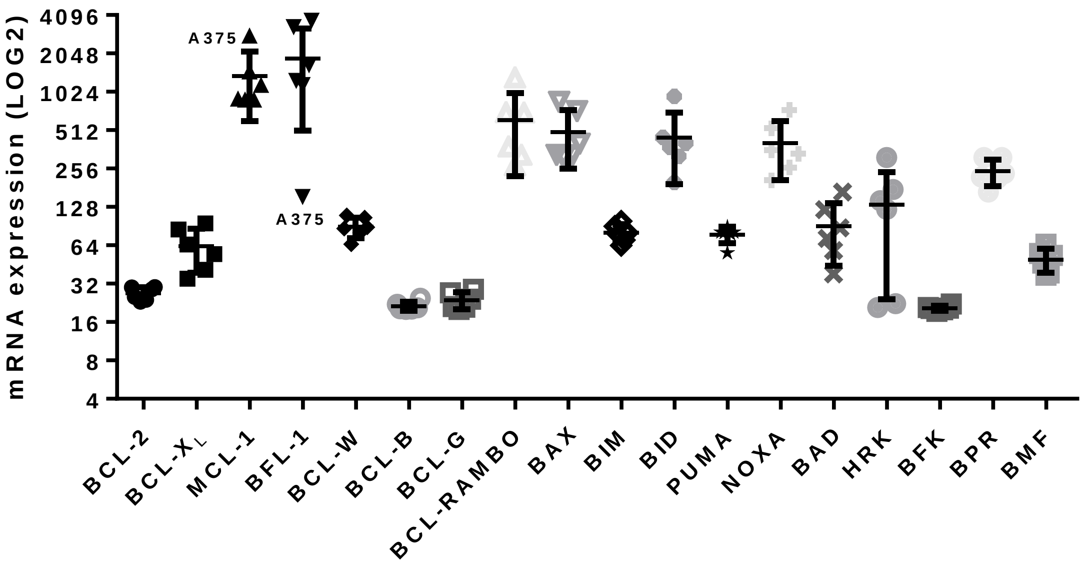

Supplementary figure 13: Overlay of BCL-2 gene expression and dependencies in SKCM cell lines. A, Heatmap of BCL-2 gene dependencies in SKCM. Percentage viability loss from all permutations of BCL-2, BCL-X_L and MCL-1 inhibitor combinations are depicted on a scale from 0% (blue) to 100% (red). **B,** mRNA expression data (LOG2 scores) from select BCL-2 genes from each SKCM cell line. Outlier cell lines are annotated by text.

Supplementary figure 14

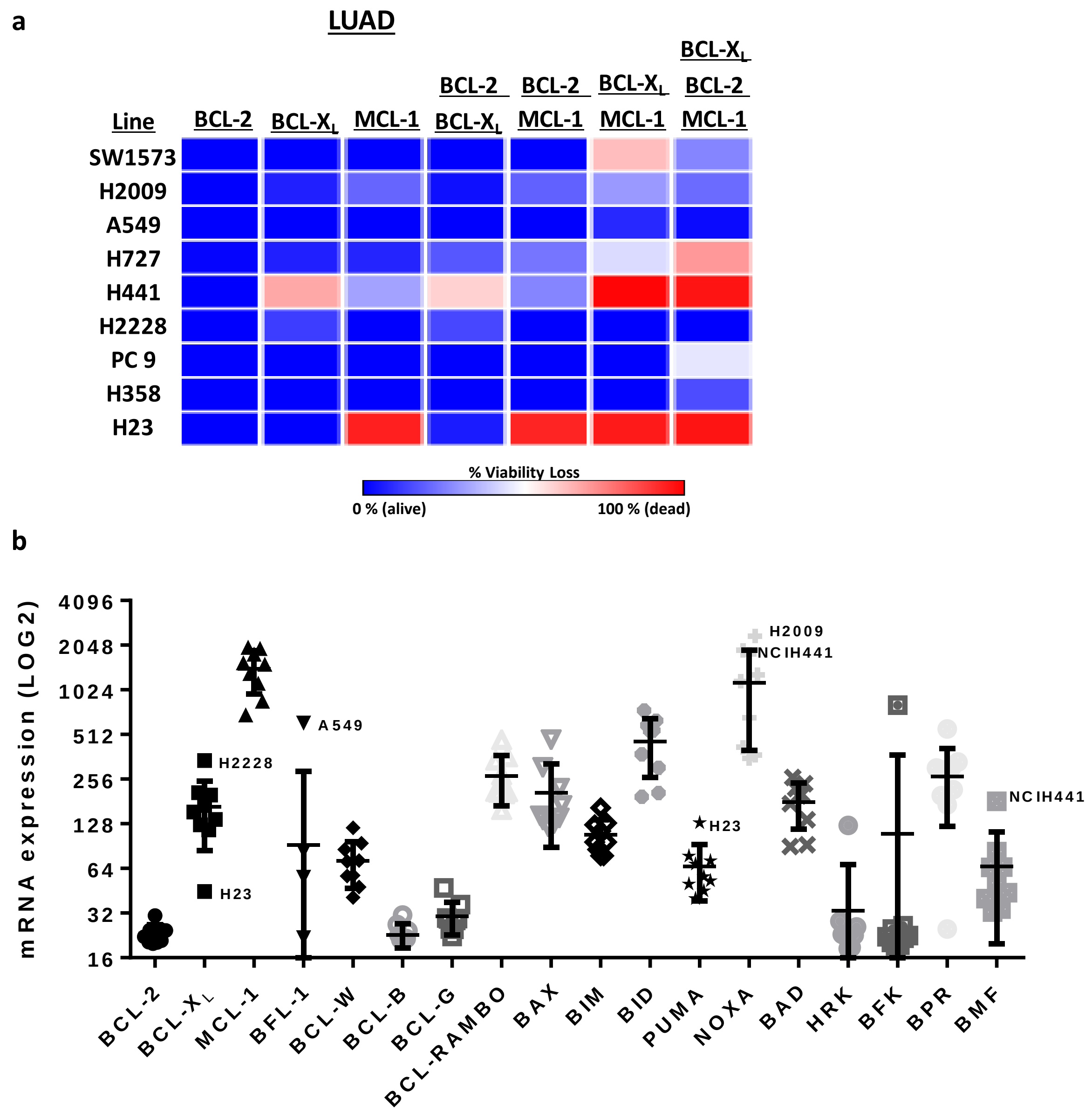

Supplementary figure 14: Overlay of BCL-2 gene expression and dependencies in LUAD cell lines. A, Heatmap of BCL-2 gene dependencies in LUAD. Percentage viability loss from all permutations of BCL-2, BCL-X_L and MCL-1 inhibitor combinations are depicted on a scale from 0% (blue) to 100% (red). **B,** mRNA expression data (LOG2 scores) from select BCL-2 genes from each LUAD cell line. Outlier cell lines are annotated by text.

Supplementary figure 15

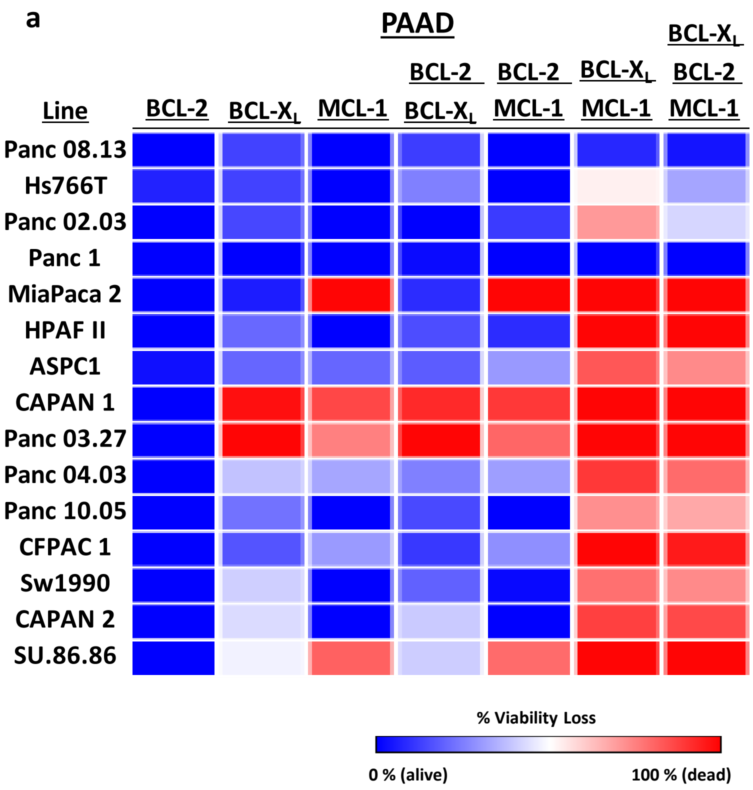

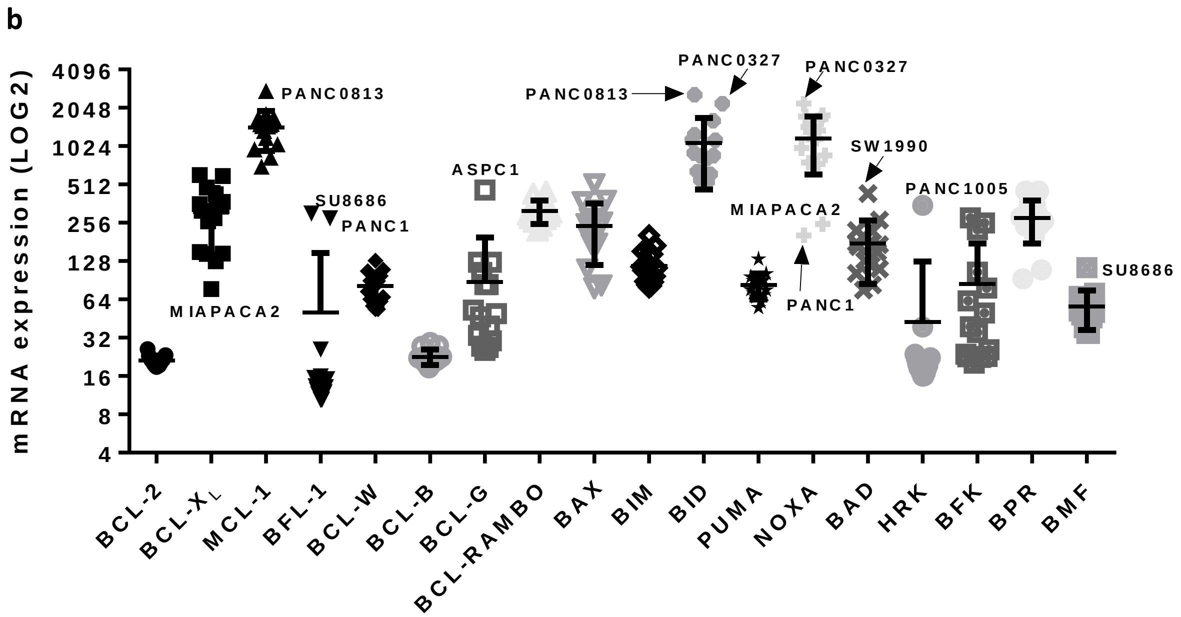

Supplementary figure 15: Overlay of BCL-2 gene expression and dependencies in PAAD cell lines. A, Heatmap of BCL-2 gene dependencies in PAAD. Percentage viability loss from all permutations of BCL-2, BCL-X_L and MCL-1 inhibitor combinations are depicted on a scale from 0% (blue) to 100% (red). **B,** mRNA expression data (LOG2 scores) from select BCL-2 genes from each PAAD cell line. Outlier cell lines are annotated by text.

Supplementary figure 16: Western blots from Figure 2

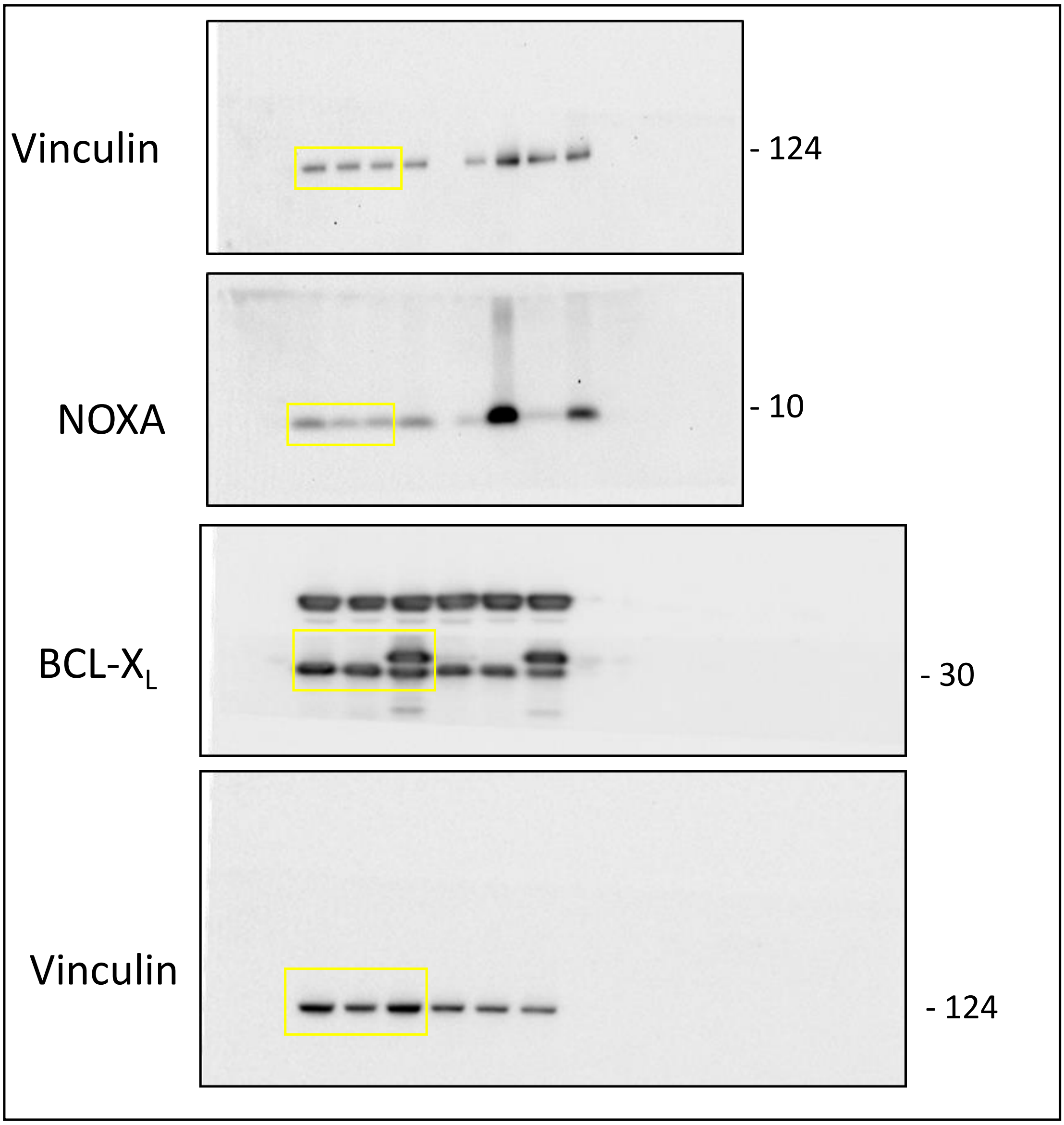

Supplementary figure 17: Western blots from Figure 3

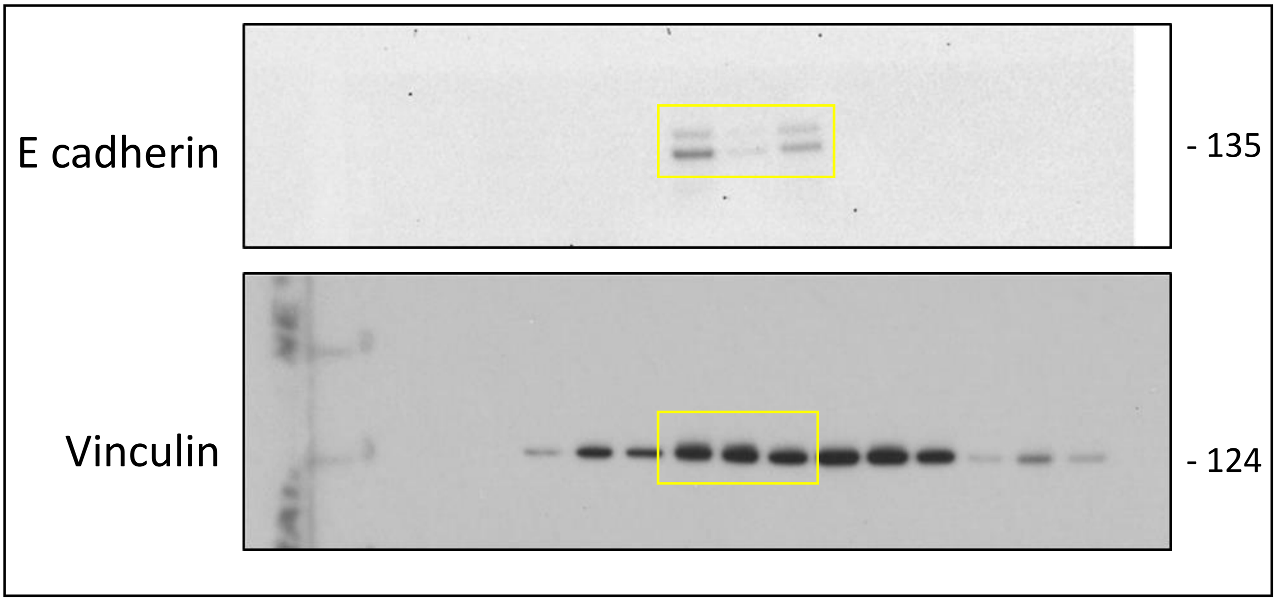

Supplementary figure 18: Western blots from Figure 5a

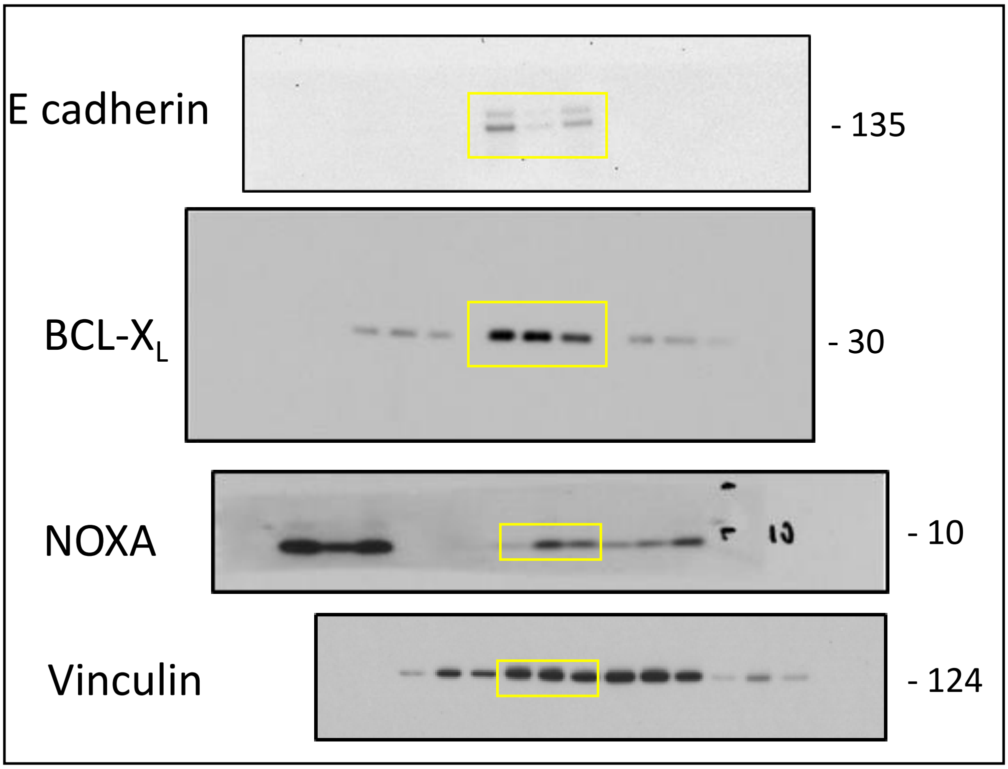

Supplementary figure 19: Western blots from Figure 5b

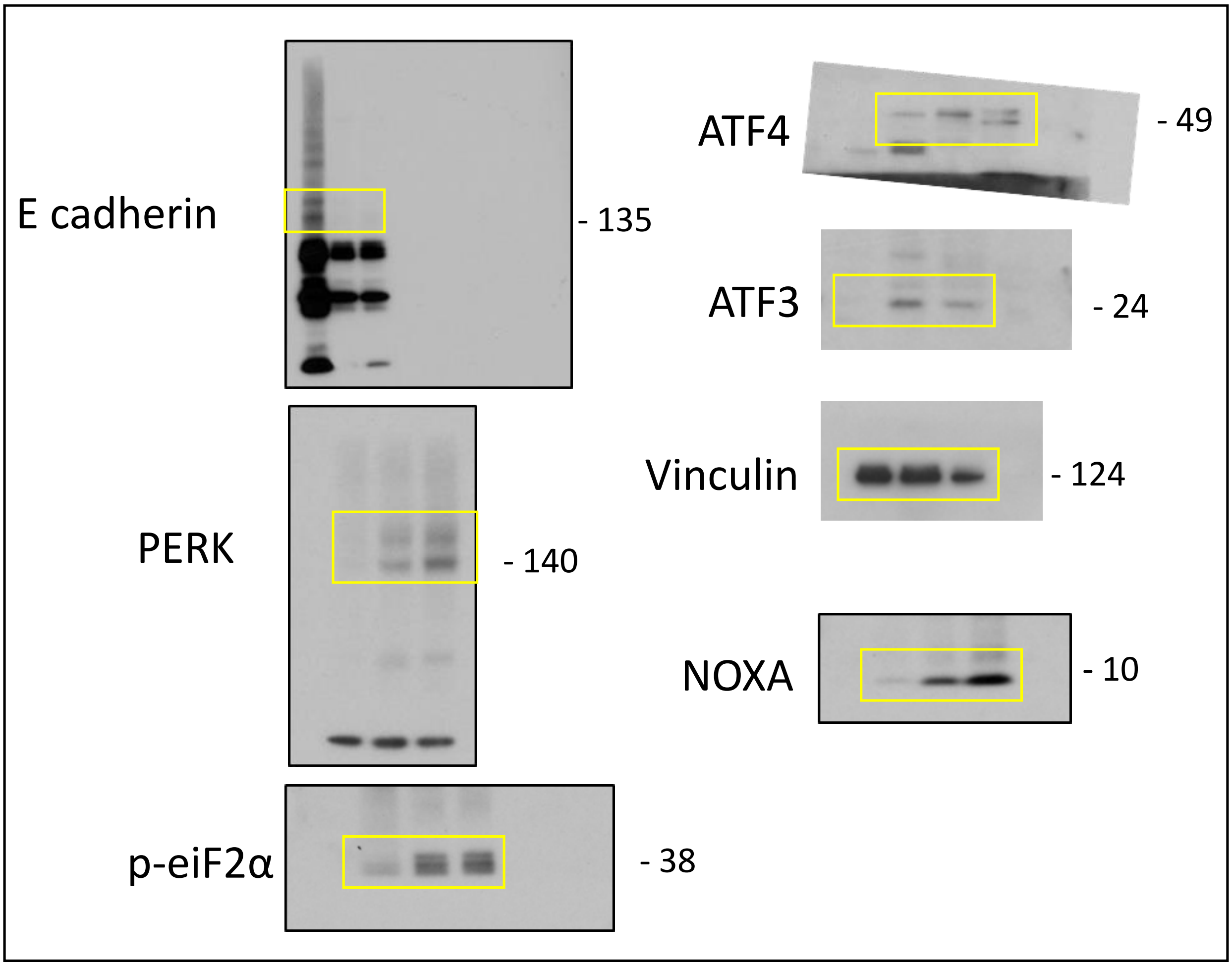

Supplementary figure 20: Western blots from Figure 5c

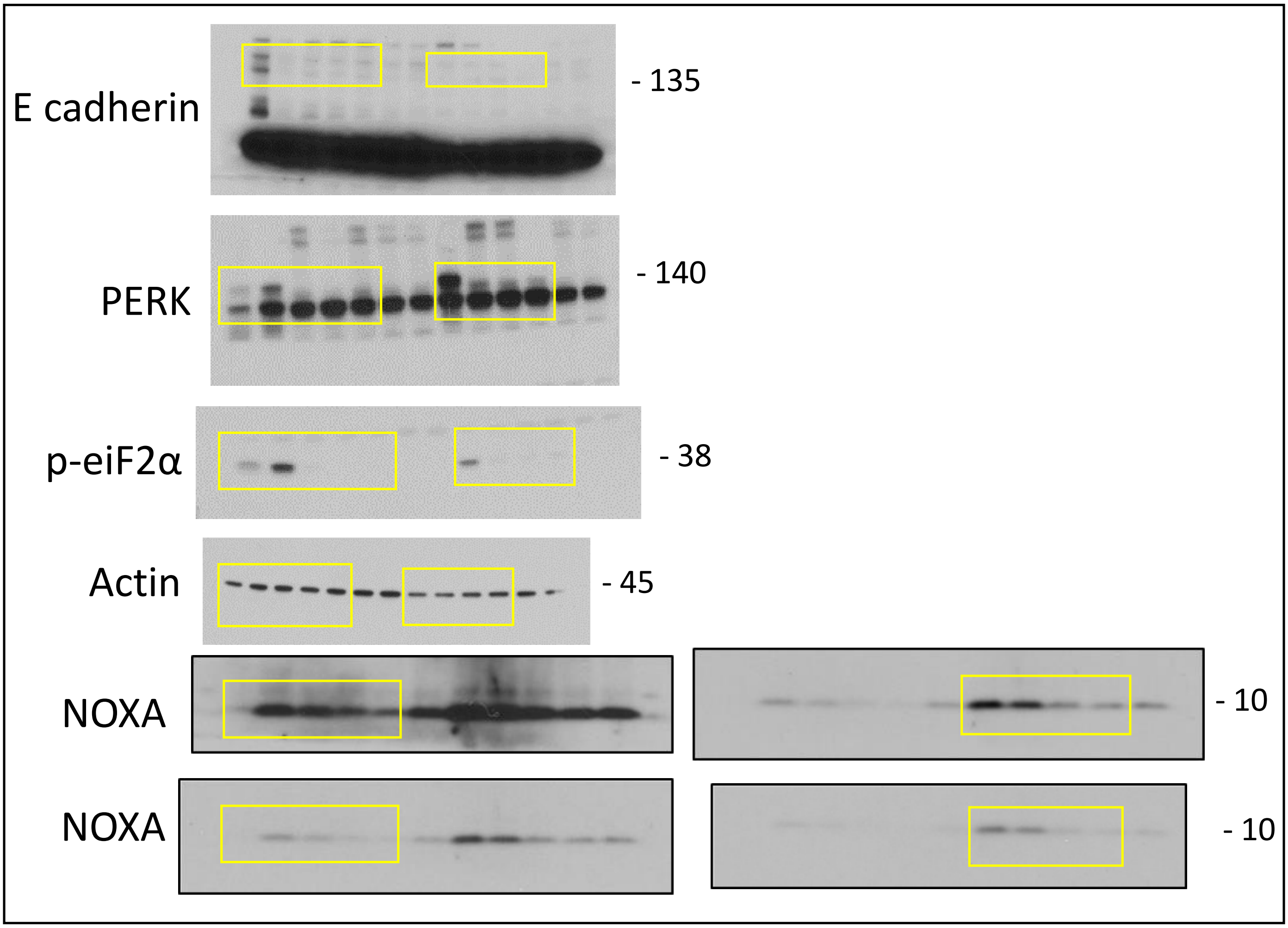

Supplementary figure 21: Westerns from Figure 6

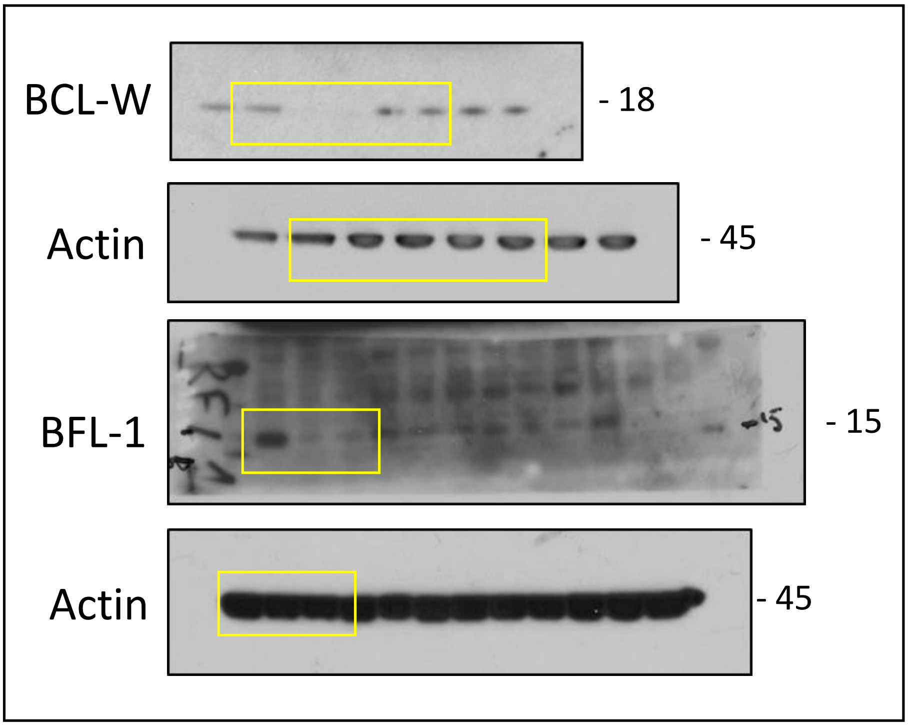

Supplementary figure 22: Westerns from Supplemental Figure 5

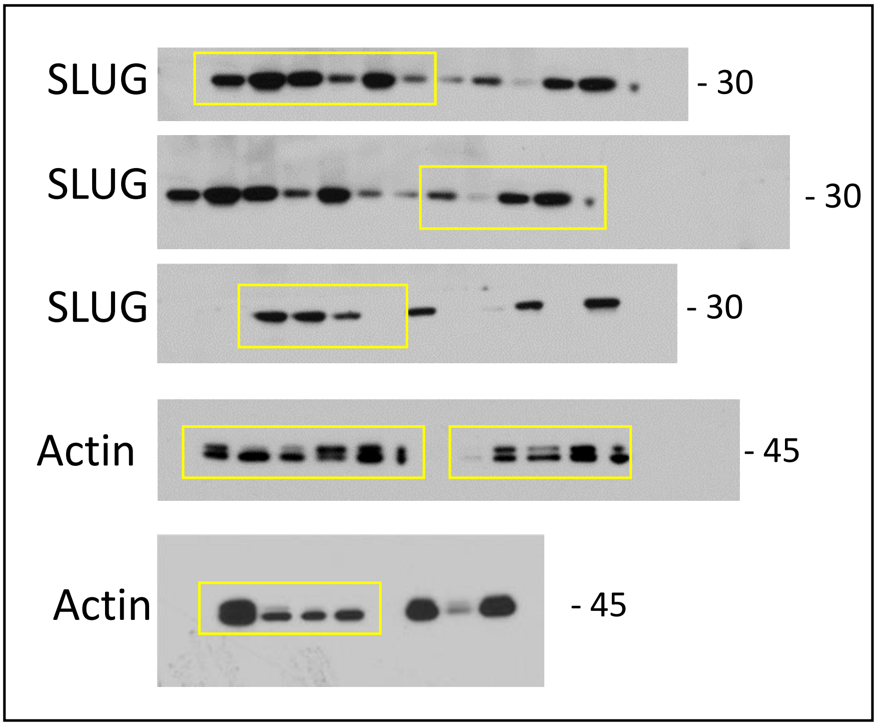

Supplementary figure 16-22: Uncropped western blots. Uncropped western blots from each figure are shown. Markers indicate the molecular weight (in kilodaltons) of each protein. Yellow boxes highlight the regions selected for the main figures.
