# Supplementary material for: Systematic mapping of BCL-2 gene dependencies in cancer reveals molecular determinants of BH3 mimetic sensitivity
Source: Nat Commun. 2018 Aug 29;9:3513. doi: 10.1038/s41467-018-05815-z (PMC6115427; doi:10.1038/s41467-018-05815-z)
Supplement: Supplementary file 3 — Description of Additional Supplementary Files [file 41467_2018_5815_MOESM3_ESM.pdf]

Description of Additional Supplementary Files

File Name: Supplementary Data 1
Details: List of all cell lines used in study

File Name: Supplementary Data 2
Details: Linear regression analysis to determine if tissue of origin or mutation status better predicts BCL-2 gene dependencies.

File Name: Supplementary Data 3
Details: BCL-2 family gene expression correlates of BCL-2, BCL-X_L, or MCL-1 dependency. A linear regression analysis was performed to compare the BCL-2 gene expression data to BCL-2, BCL-X_L, or MCL-1 dependencies. The effect size and p-values are plotted here.

File Name: Supplementary Data 4
Details: Results from a pooled negative-selection CRISPR screen for sensitizers to BCL-X_L + MCL-1 co-inhibition in the resistant cell line TYKNU. The CRISPR library is shown, as well as the depletion scores (based on the 3-scores) for each replicate. Genes are then ranked based on average depletion scores from the 2 replicates, and the genes shown in red were selected for further validation.

File Name: Supplementary Data 5
Details: Phenotypic outlier cell lines have outlying expression levels of response modifying BCL-2 genes. Fifteen cell lines from 9 different tissue types were noted to have BCL-2 gene dependency data that stood out as unique compared to the other cell lines from the same tissue. In addition, the expression levels of certain BCL-2 genes from these cell lines were also unique compared to the average expression levels from the same tissues. The unique phenotypes and possible molecular explanations are included in the table.

File Name: Supplementary Data 6
Details: List of all shRNA and CRISPR sequences used in study.
